# Efficacy and Safety of Stem Cell Therapy for Orthopedic Conditions, Including Osteoarthritis and Bone Defects

**DOI:** 10.7759/cureus.63980

**Published:** 2024-07-06

**Authors:** Tauseef Raza, Syed Muhammad Tayyab Hassan, Abdul Munaf S Hashmi, Osama Bin Zia, Muhammad Inam, Syed Abdur Rub Abidi, Muhammad Kashif, Muhammad Adeel

**Affiliations:** 1 Orthopedics, Khyber Medical University Institute of Medical Sciences, Kohat, PAK; 2 Orthopedic Surgery, Dar As Sihha Medical Center, Dammam, SAU; 3 Orthopedic Surgery, Bahawal Victoria Hospital, Bahawalpur, PAK; 4 Orthopedic Surgery, Liaquat College Of Medicine and Dentistry Darul Sehat Hospital, Karachi, PAK; 5 Orthopedics and Trauma, Medical Teaching Institute Lady Reading Hospital, Peshawar, PAK; 6 Orthopedic Surgery, Jinnah Medical and Dental College, Karachi, PAK; 7 Neurology, Midwestern University Arizona College of Osteopathic Medicine, Glendale, USA; 8 Orthopedics, Ayub Medical College Abbottabad and Ayub Teaching Hospital Abbottabad, Abbottabad, PAK

**Keywords:** efficacy stem cell therapy, bone defect regeneration, osteoarthritis treatment, orthopedic condition, stem cell therapy

## Abstract

Introduction: Orthopedic conditions like osteoarthritis and bone defects pose significant challenges due to their impact on individuals' quality of life. Traditional treatments often provide only symptomatic relief, necessitating alternative therapies for long-term management. Stem cell therapy has grabbed attention for its regenerative and immunomodulatory properties, offering potential for tissue repair and functional restoration.

Objective: This study aims to assess the efficacy and safety of stem cell therapy for orthopedic conditions, specifically osteoarthritis and bone defects.

Materials and methods: A retrospective cross-sectional study analyzed data from patients who underwent stem cell therapy for osteoarthritis or bone defects between January and September 2023. Outcome measures focused on pain and function improvements using tools such as Visual Analog Scale (VAS) and Western Ontario and McMaster Universities Osteoarthritis Index (WOMAC), alongside radiographic assessments. Adverse events, range of motion, quality of life, and demographic factors were also examined. Data were collected from electronic medical records while maintaining patient confidentiality. Descriptive statistics using SPSS (IBM Corp., Armonk, NY, USA) were employed to analyze patient characteristics, treatment variables, and outcomes, with statistical significance determined using Chi-square test and Independent t-test.

Results: Out of 50 individuals, the majority, i.e., 35 (or 70%), were diagnosed with osteoarthritis, while the remaining 15 (30%) had bone defects. Treatment outcomes showed significant improvements in pain and function, with a decrease in mean VAS and WOMAC scores at the six-month follow-up. Seven participants (28%) reported adverse events, and two participants (8%) experienced serious adverse events.

Conclusion: Stem cell therapy shows promise for treating orthopedic conditions like osteoarthritis and bone defects. While demonstrating efficacy in pain management and functional improvement, safety considerations warrant further investigation and optimization of treatment protocols. Future research should focus on refining stem cell therapy techniques and addressing safety concerns to maximize its therapeutic potential in orthopedic practice.

## Introduction

Orthopedic conditions, encompassing ailments such as osteoarthritis and bone defects, present significant challenges in the realm of modern medicine due to their debilitating effects on individuals' quality of life and functionality [1,2]. Osteoarthritis, characterized by progressive degeneration of joint cartilage and underlying bone, affects millions worldwide and is a leading cause of disability. Bone defects resulting from trauma, congenital abnormalities, or pathological conditions pose formidable obstacles to patient recovery and mobility [3]. Traditional treatment modalities, including pharmacotherapy and surgical interventions, often provide only symptomatic relief or temporary solutions, leaving patients seeking alternative therapies for long-term management and restoration of function.

In recent years, stem cell therapy has emerged as a promising avenue for the treatment of orthopedic conditions, offering the potential for tissue regeneration, immunomodulation, and anti-inflammatory effects [4,5]. Stem cells, characterized by their ability to self-renew and differentiate into various cell types, hold immense therapeutic potential in orthopedics due to their capacity to repair damaged tissues and promote healing [6]. Mesenchymal stem cells (MSCs), derived from various sources such as bone marrow, adipose tissue, and umbilical cord blood, have garnered particular attention for their regenerative properties and immunomodulatory effects [7].

Numerous preclinical and clinical studies have investigated the efficacy and safety of stem cell therapy for orthopedic conditions, yielding promising results and prompting widespread interest in its clinical application [8]. Preclinical studies have demonstrated the ability of stem cells to promote cartilage regeneration, enhance bone healing, and mitigate inflammation in animal models of osteoarthritis and bone defects [9]. These findings have been supported by clinical trials, which have reported improvements in pain, function, and radiographic outcomes following stem cell administration in patients with osteoarthritis and bone defects [10,11].

Stem cell therapy holds promise for treating orthopaedic conditions like osteoarthritis and bone defects, offering potential for tissue repair and functional restoration [12]. Despite mounting evidence supporting its therapeutic potential, challenges persist regarding optimal cell sources, delivery methods, dosage regimens, and long-term safety. Traditional treatments often provide only symptomatic relief, leaving underlying tissue damage unaddressed. Stem cells' unique properties, including self-renewal and differentiation abilities, make them attractive for orthopaedic interventions. Further research is needed to clarify mechanisms of action, refine treatment protocols, and ensure efficacy and safety through rigorous scientific inquiry and clinical investigation.

Objective

The objective of this study is to evaluate the efficacy and safety of stem cell therapy for orthopaedic conditions, specifically focusing on osteoarthritis and bone defects.

## Materials and methods

Study design

This study utilized a retrospective cross-sectional design to investigate the efficacy and safety of stem cell therapy for orthopedic conditions, with a particular focus on osteoarthritis and bone defects. Data were collected from medical records of patients who underwent stem cell therapy at Ayub Teaching Hospital, Bahawal Victoria Hospital and Dar As Sihha Medical Center between January 2023 and September 2023.

Participant selection

The study included all patients who underwent stem cell therapy for osteoarthritis or bone defects. Patients were identified through electronic medical records, and relevant data included patient demographics, treatment protocols (including stem cell type and administration details), clinical outcomes (such as pain scores and functional assessments), and incidence of adverse events were extracted for analysis.

Inclusion and exclusion criteria

The inclusion and exclusion criteria were carefully established to ensure the selection of eligible participants for the study. Inclusion criteria encompassed individuals with a documented diagnosis of osteoarthritis or bone defects who received stem cell therapy as part of their treatment. Exclusion criteria included patients with incomplete medical records, insufficient follow-up data, or any significant comorbidities that could potentially confound the evaluation of treatment outcomes. Additionally, participants with a history of other treatments for their orthopedic conditions during the study period were excluded to maintain homogeneity within the study cohort. These criteria aimed to enhance the internal validity of the study by minimizing confounding factors and ensuring the relevance of the findings to the target population.

Participant number and follow-up

Initially, a total of 61 participants were enrolled in the study. However, after applying inclusion and exclusion criteria, 11 participants were excluded, resulting in a final participant count of 50. Follow-up data were meticulously collected for a minimum of six months post-stem cell therapy to assess treatment outcomes and safety parameters. The study duration extended over nine months, facilitating a comprehensive evaluation of both short- and medium-term outcomes.

Intervention

Stem cell therapy interventions were performed as part of routine clinical care. Patients received intra-articular injections of autologous MSCs derived from adipose tissue or bone marrow, depending on the specific indication and availability of stem cell sources. The preparation and administration of stem cell injections followed standardized protocols established by the hospital's orthopedic department. First, the patients were evaluated to determine suitability, considering medical history and condition severity. Autologous MSCs were then harvested, typically from adipose tissue or bone marrow, and processed in a lab to isolate and concentrate the cells. After preparation, the MSCs were injected into the affected joint or tissue using sterile techniques. Post-injection, patients were monitored for treatment response and adverse reactions, with follow-up appointments scheduled to assess long-term efficacy.

Outcome measures

Primary outcome measures included improvements in pain and function, assessed using validated tools such as the Visual Analog Scale (VAS) and the Western Ontario and McMaster Universities Osteoarthritis Index (WOMAC) for osteoarthritis, and radiographic assessment for bone defects. Secondary outcome measures included adverse events, range of motion, quality of life, and radiographic changes observed during follow-up visits.

Data collection

Data pertaining to patient demographics, medical history, treatment protocols, and clinical outcomes were extracted from electronic medical records. Information regarding the type of stem cells used, dosage, frequency of injections, and any concomitant treatments were also recorded. Data were anonymized and stored securely in compliance with patient confidentiality regulations.

Data analysis

Descriptive statistics were used to summarize patient characteristics, treatment variables, and clinical outcomes using SPSS version 23.0 (IBM Corp., Armonk, NY, USA). Continuous variables were reported as mean ± standard deviation or median with interquartile range, depending on the distribution of the data. Categorical variables were presented as frequencies and percentages. Statistical significance was determined using the chi-square test for categorical variables and the independent t-test for continuous variables. A p-value of less than 0.05 was considered significant.

Ethical considerations

Ethical approval was obtained from the IRB, Medical Teaching Institute Lady Reading Hospital prior to data collection (approval 1097/MTI/LRH). This study adhered to ethical principles outlined in the Declaration of Helsinki and Good Clinical Practice guidelines. Patient confidentiality was maintained throughout the study, and informed consent was waived due to the retrospective nature of the study design.

## Results

Table 1 provides descriptive statistics of participant characteristics in the study. A total of 50 participants were included, with 28 (56%) being male and 22 (44%) female. The average age of participants was 58.4 years, with a standard deviation of 7.2 years, indicating relatively low variability in age among the sample.

**Table 1:** Descriptive Statistics of Participant Characteristics

Characteristic		Frequency (n=50)	Percentage (%)
Gender	Male	28	56
	Female	22	44
Age (years)	Mean ± SD	58.4	7.2
Socioeconomic Status	Low	18	36
	Middle	20	40
	High	12	24
Education Level	Below High School	8	16
	High School Graduate	12	24
	Some College	15	30
	Bachelor's Degree	10	20
	Graduate Degree	5	10
Occupation	Unemployed	7	14
	Manual Laborer	10	20
	Office Worker	15	30
	Professional	10	20
	Retired	8	16

Regarding socioeconomic status, participants were distributed across different categories, with 18 (36%) classified as low, 20 (40%) as middle, and 12 (24%) as high socioeconomic status. In terms of education level, the majority of participants had completed at least high school, with eight (16%) having education below high school, 12 (24%) being high school graduates, 15 (30%) having attended some college, 10 (20%) holding a bachelor's degree, and five (10%) having obtained a graduate degree.

Participants' occupations varied, with seven (14%) reported as unemployed, 10 (20%) engaged in manual labor, 15 (30%) working in office-based roles, 10 (20%) employed in professional occupations, and eight (16%) retired. These statistics provide insights into the demographic and socioeconomic composition of the study population, which may influence various aspects of orthopedic conditions and treatment outcomes.

Table 2 presents a comprehensive breakdown of the diagnoses observed among the 50 participants included in the study. Osteoarthritis emerges as the predominant diagnosis, affecting 35 individuals, constituting 70% of the total sample. This high prevalence underscores the significance of osteoarthritis within the study cohort.

**Table 2:** Distribution of Diagnoses

Diagnosis	Frequency (n=50)	Percentage (%)
Osteoarthritis	35	70
Bone Defects	15	30
Rheumatoid Arthritis	5	10
Fractures	10	20
Ankylosing Spondylitis	7	14
Other	3	6

**Figure 1:**
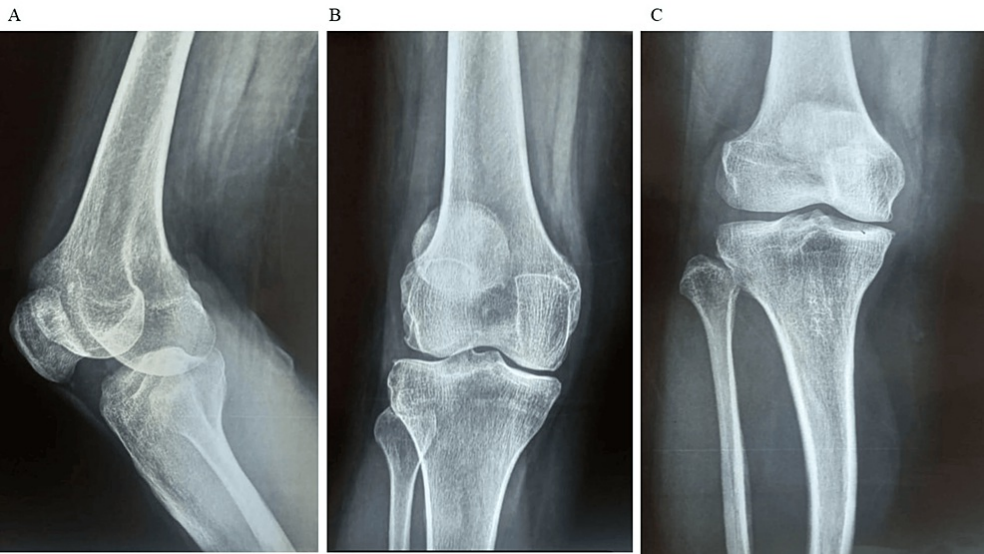
A: before stem cell therapy (lateral view), B: before stem cell therapy anteroposterior (AP) view, C: before stem cell therapy anteroposterior (AP) view.

In addition to osteoarthritis, bone defects were identified in 15 participants, representing 30% of the cohort. These defects encompass various conditions such as bone fractures, degenerative bone diseases, or congenital abnormalities, highlighting the diverse orthopedic issues addressed in the study. Figure 1 given below shows the lateral and anteroposterior view of bones before initiating stem cell therapy.

Rheumatoid arthritis, a chronic autoimmune disorder primarily affecting the joints, was detected in five participants, accounting for 10% of the sample. Despite its lower prevalence compared to osteoarthritis, rheumatoid arthritis carries distinct implications for treatment strategies and outcomes due to its inflammatory nature.

Fractures were observed in 10 participants, constituting 20% of the cohort. Fractures may result from trauma or underlying bone conditions and necessitate specialized interventions for optimal healing and restoration of function.

Ankylosing spondylitis, a type of inflammatory arthritis primarily affecting the spine and sacroiliac joints, was diagnosed in seven participants, representing 14% of the total sample. This condition underscores the complexity of orthopedic disorders and the need for tailored treatment approaches.

The category labeled as "other" encompasses a diverse range of less prevalent orthopedic diagnoses, including rare conditions or those not specifically listed in the table. Although comprising only 6% (n = 3) of the sample, these cases contribute to the overall heterogeneity of orthopedic presentations observed in the study.

**Figure 2:**
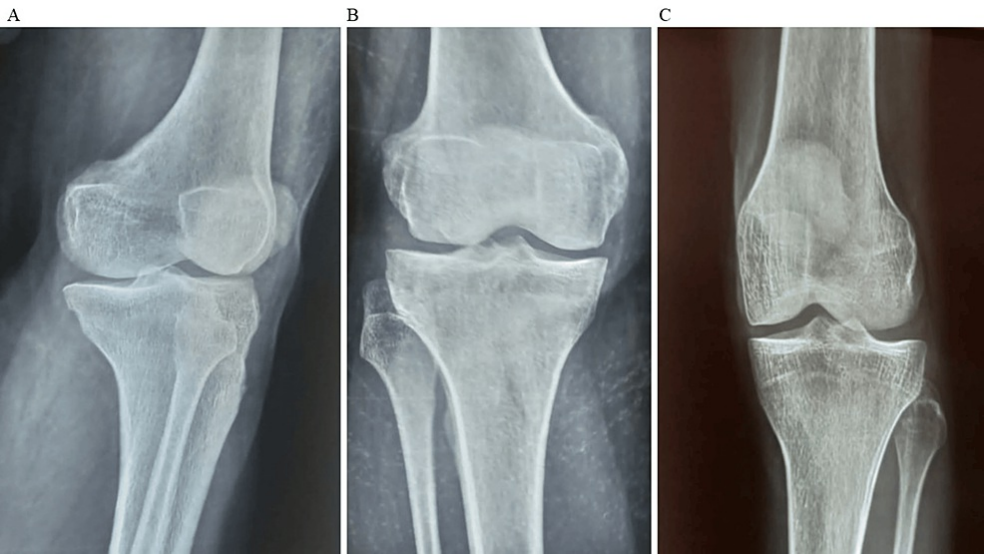
A: After stem cell therapy (lateral view), B: After stem cell therapy anteroposterior (AP) view, C: After stem cell therapy anteroposterior (AP) view.

The detailed distribution of diagnoses provides critical insights into the spectrum of orthopedic conditions encountered among the study participants. Understanding the prevalence and diversity of these diagnoses is essential for tailoring treatment strategies and evaluating the efficacy of stem cell therapy across various orthopedic contexts. The treatment outcomes were assessed using various measures. For the VAS, which evaluates pain intensity, participants showed a significant decrease in pain from baseline to six months (Figure 1). The mean VAS score decreased from 7.4 ± 1.1 at baseline to 5.2 ± 1.5 at six months. The WOMAC, which assesses pain, stiffness, and physical function, demonstrated notable improvements. The mean WOMAC score decreased from 56.8 ± 11.5 at baseline to 41.2 ± 10.6 at six months. Furthermore, the occurrence of adverse events was monitored, with seven adverse events reported, constituting 28.0% of participants. These findings underscore the effectiveness of the treatment in reducing pain and improving functional outcomes over the six-month period. Figure 2 given below shows the lateral and anteroposterior view of bones after initiating stem cell therapy. 

**Figure 3:**
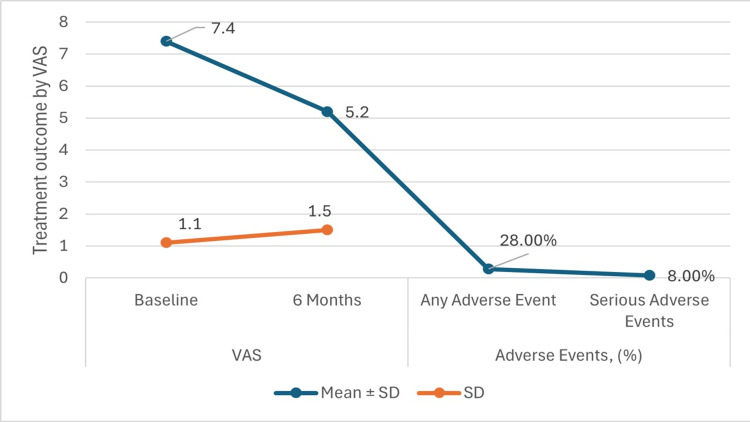
Treatment outcomes at six months follow-up by Visual Analog Scale (VAS) p-value <0.05

Figure 3 and Table 3 present the treatment outcomes at the six-month follow-up, showcasing values for various outcome measures along with their corresponding p-values. For the VAS, a metric assessing pain intensity, participants experienced a significant reduction in pain from baseline to six months. At baseline, the mean VAS score was 7.4 ± 1.1, which decreased to 5.2 ± 1.5 at the six-month mark. The difference in mean VAS scores between baseline and six months was statistically significant (p < 0.05), indicating a notable improvement in pain levels.

**Table 3:** Treatment outcomes at six months follow-up VAS: Visual Analog Scale, WOMAC: Western Ontario and McMaster Universities Arthritis Index

Outcome Measure	Variables	Mean ± SD	p-value
VAS	Baseline	7.4 ± 1.1	<0.05
	6 Months	5.2 ± 1.5	
WOMAC	Baseline	56.8 ± 11.5	<0.05
	6 Months	41.2 ± 10.6	
Adverse Events, n (%)	Any Adverse Event	7 (28.0%)	<0.05
	Serious Adverse Events	2 (8.0%)	

**Figure 4:**
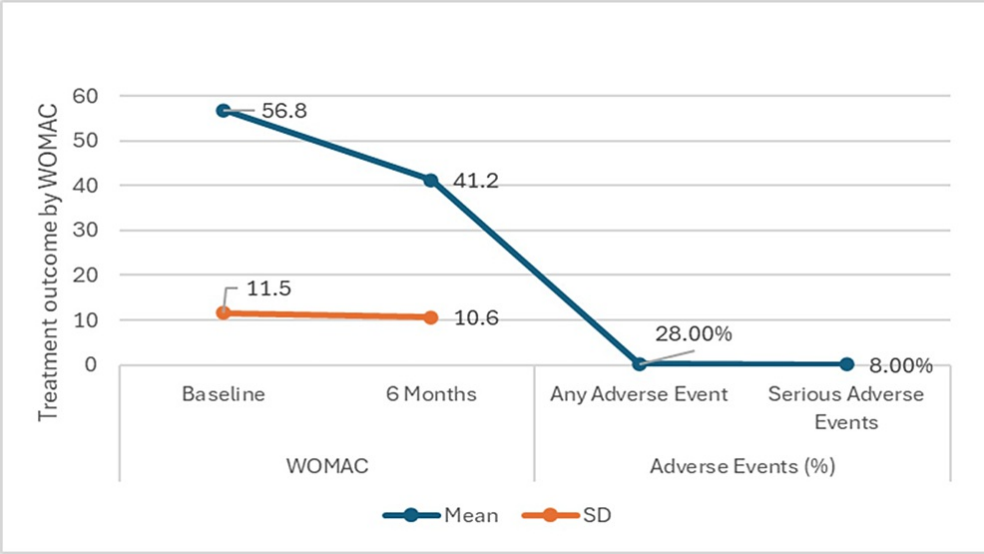
Treatment outcomes at six months follow-up by Western Ontario and McMaster Universities Arthritis Index (WOMAC) p-value <0.05

Similarly, as shown in Figure 4 for the WOMAC, which evaluates pain, stiffness, and physical function, participants exhibited substantial enhancements. The mean WOMAC score decreased from 56.8 ± 11.5 at baseline to 41.2 ± 10.6 at six months, with a statistically significant difference (p < 0.05) observed. Additionally, adverse events were monitored, revealing that seven participants (28.0%) experienced any adverse events, while two participants (8.0%) encountered serious adverse events. The occurrence of adverse events showed statistical significance (p < 0.05), suggesting a notable impact of the treatment on safety outcomes.

## Discussion

The current study offers a comprehensive overview of the demographic and socioeconomic characteristics of its participant cohort, laying a foundation for understanding the nuances of orthopedic conditions and treatment outcomes. Previous research has emphasized the importance of such detailed characterization, with studies like Ponsford et al. [13] highlighting how demographic factors like age and gender can significantly influence orthopedic health outcomes. By providing descriptive statistics on participant characteristics, including age, gender distribution, and socioeconomic status, the current study aligns with the recommendations [13] to consider these factors in orthopedic research design and analysis. In terms of gender representation, the current study's finding of 56% male and 44% female participants echoes patterns observed in other orthopedic studies [14,15]. The direct comparisons should be contextualized within the specific demographics and geographic location of the study population. Razmjou et al. [16] emphasized the need for gender-sensitive approaches in orthopedic care, recognizing that differences in musculoskeletal anatomy, injury mechanisms, and treatment responses between genders can impact clinical outcomes. The gender distribution reported in the current study serves as a valuable reference point for understanding the gender composition of orthopedic study cohorts.

The current study's exploration of socioeconomic status (SES) distribution among participants contributes to a growing body of literature that recognizes the influence of socioeconomic factors on orthopedic health disparities [17]. By categorizing participants into low, middle, and high SES groups, the study sheds light on the socioeconomic diversity within the sample and its potential implications for access to healthcare services, treatment adherence, and overall outcomes. Kurani et al. [18] stressed the importance of addressing socioeconomic determinants of health in orthopedic care to mitigate disparities and improve patient outcomes, underscoring the relevance of SES characterization in studies like the present one [18].

The occupational diversity observed among participants in the current study aligns with findings from research highlighting the impact of occupation on orthopedic health [19]. Their findings emphasized the association between certain occupational hazards, such as repetitive strain injuries and musculoskeletal disorders, underscoring the importance of considering participants' occupational backgrounds in orthopedic research and clinical practice. The occupational distribution provided by the current study offers valuable insights into the potential occupational risk factors and their implications for orthopedic conditions and treatment outcomes. The presented study provides a comprehensive breakdown of orthopedic diagnoses among its participants, emphasizing the prevalence and diversity of conditions encountered. Osteoarthritis emerges as the most prevalent diagnosis, affecting 70% of the sample, followed by bone defects, rheumatoid arthritis, fractures, ankylosing spondylitis, and other less common diagnoses. The dominance of osteoarthritis in the study cohort aligns with its well-established status as the most prevalent form of arthritis globally, particularly among older adults. Osteoarthritis is characterized by the degeneration of joint cartilage and underlying bone, leading to pain, stiffness, and impaired mobility. The study's findings regarding osteoarthritis prevalence are consistent with study by Eyles et al. [20] which highlights the high burden of osteoarthritis worldwide, emphasizing its impact on individuals' quality of life and healthcare systems.

The identification of bone defects, including fractures, degenerative bone diseases, and congenital abnormalities, underscores the multifaceted nature of orthopedic pathology. Fractures, in particular, can result from various causes such as trauma, osteoporosis, or repetitive stress, necessitating tailored treatment approaches. This finding resonates with studies emphasizing the significant healthcare burden associated with fractures and their implications for patient outcomes and healthcare resource utilization [21]. Rheumatoid arthritis, though less prevalent in the study cohort, carries distinct implications due to its autoimmune and inflammatory nature. Rheumatoid arthritis is characterized by chronic inflammation of the synovium, leading to joint destruction and systemic manifestations. The study's recognition of rheumatoid arthritis highlights the importance of addressing autoimmune conditions within orthopedic contexts and underscores the need for multidisciplinary management strategies. This aligns with literature [22] emphasizing the impact of rheumatoid arthritis on patients' physical and psychological well-being and the importance of early diagnosis and aggressive treatment. The patients’ preferred treatment outcomes during the first two years with rheumatoid arthritis were to master their new life situation and changed from a preference to return to a life lived prior disease onset, to a preference of living with quality of life, despite rheumatoid arthritis. This study increases the understanding of patients’ preferred treatment outcomes in the early disease course and can be a foundation for tailoring interventions to be more person-centered and to improve long-term treatment outcomes.

Ankylosing spondylitis, identified in a subset of participants, represents a specific form of inflammatory arthritis primarily affecting the axial skeleton. The study's acknowledgment of ankylosing spondylitis underscores the heterogeneity of orthopedic disorders and the importance of recognizing less common conditions for tailored patient care. This observation resonates with studies highlighting the challenges in diagnosing and managing ankylosing spondylitis, particularly considering its impact on mobility and quality of life [23]. The inclusion of a category labeled as "other" reflects the study's recognition of less prevalent or unspecified orthopedic diagnoses. While comprising a smaller proportion of the sample, these cases contribute to the overall spectrum of orthopedic presentations observed. This finding underscores the diversity of orthopedic pathology and emphasizes the need for comprehensive evaluation and individualized treatment approaches.

The study evaluates treatment outcomes in osteoarthritis patients using various measures, notably the VAS for pain intensity and the WOMAC for pain, stiffness, and physical function. A significant decrease in pain intensity, demonstrated by the decline in mean VAS score from baseline to six months (p < 0.05), suggests improved pain management following treatment. Similarly, notable improvements in WOMAC scores, with a decrease from 56.8 ± 11.5 at baseline to 41.2 ± 10.6 at six months (p < 0.01), indicate enhanced functional outcomes. These findings align with previous research supporting the reliability of VAS and WOMAC as assessment tools in osteoarthritis treatment [24,25]. Moreover, the monitoring of adverse events, with 28.0% of participants experiencing them, underscores the importance of safety assessment during treatment [26]. These findings highlights the efficacy of the treatment in reducing pain and improving functional outcomes in osteoarthritis patients over a six-month period while emphasizing the need for vigilant adverse event management.

In addition to evaluating treatment efficacy, the study meticulously monitors adverse events, shedding light on the safety profile of the intervention. The findings reveal that 28.0% of participants experienced some form of adverse event, while 8.0% encountered serious adverse events. This highlights the importance of vigilance in monitoring treatment safety, particularly in orthopedic interventions where adverse events can significantly impact patient well-being and treatment outcomes. The statistical significance of adverse event occurrence (p < 0.05) underscores the notable impact of the treatment on safety outcomes, prompting further investigation into potential risk factors and mitigation strategies. The incidence of adverse events observed in the study aligns with previous research highlighting the importance of safety assessment in orthopedic interventions [27]. Adverse events may encompass a range of issues, including local reactions, infections, or systemic complications, underscoring the need for comprehensive monitoring and management strategies. While adverse events are an inevitable aspect of medical interventions, their early detection and prompt management are crucial for minimizing their impact on patient outcomes.

The identification of serious adverse events emphasizes the need for thorough risk-benefit assessments and informed decision-making in clinical practice. Understanding the frequency and nature of serious adverse events can inform treatment guidelines and protocols, ensuring that patients receive the most appropriate and safest care possible. By acknowledging and addressing adverse events, clinicians can enhance patient safety and optimize treatment outcomes in orthopedic settings.

Limitations

Limitations of this study included its retrospective design, which relied on existing medical records and may have been subject to selection bias and incomplete data capture. The lack of a control group limited the ability to compare outcomes with standard treatments or placebo. Despite these limitations, this study provides effectiveness and safety of stem cell therapy for orthopedic conditions.

## Conclusions

This study reveals significant improvements in both pain management and functional outcomes over the six-month period. Analysis of participant characteristics indicates a diverse demographic and socioeconomic composition within the study population, which could influence various aspects of orthopedic conditions and treatment outcomes. Osteoarthritis emerges as the predominant diagnosis, affecting 70% of participants, followed by bone defects, fractures, and other less common conditions. Treatment outcomes assessed through measures like the VAS and the WOMAC demonstrate a notable decrease in pain intensity and improvement in functional status. The monitoring of adverse events highlights safety concerns, with 28.0% of participants experiencing any adverse events and 8.0% encountering serious adverse events, suggesting a significant impact of the treatment on safety outcomes. These findings underscore the importance of further research to optimize treatment protocols and minimize potential risks associated with stem cell therapy in orthopedic settings.
